# Investigating Pathogenicity and Virulence of *Staphylococcus pettenkoferi:* An Emerging Pathogen

**DOI:** 10.3390/ijms222413614

**Published:** 2021-12-19

**Authors:** Nour Ahmad-Mansour, Lucile Plumet, Sylvaine Huc-Brandt, Chloé Magnan, Alex Yahiaoui-Martinez, Karima Kissa, Alix Pantel, Jean-Philippe Lavigne, Virginie Molle

**Affiliations:** 1 Laboratory of Pathogen Host Interactions, Université de Montpellier, CNRS, UMR 5235, 34095 Montpellier, France; nour.mansour@umontpellier.fr (N.A.-M.); lucile.plumet@gmail.com (L.P.); sylvaine.huc-brandt@umontpellier.fr (S.H.-B.); karima.kissa-marin@umontpellier.fr (K.K.); 2 Virulence Bactérienne et Infections Chroniques, INSERM U1047, Department of Microbiology and Hospital Hygiene, CHU Nîmes, Université de Montpellier, 30908 Nimes, France; chloe.magnan@live.fr (C.M.); alex.yahiaouimartinez@chu-nimes.fr (A.Y.-M.); alix.pantel@chu-nimes.fr (A.P.); jean.philippe.lavigne@chu-nimes.fr (J.-P.L.)

**Keywords:** *Staphylococcus pettenkoferi*, emerging pathogen, virulence, persistence, zebrafish infections

## Abstract

*Staphylococcus pettenkoferi* is a coagulase-negative *Staphylococcus* identified in 2002 that has been implicated in human diseases as an opportunistic pathogenic bacterium. Its multiresistant character is becoming a major health problem, yet the pathogenicity of *S. pettenkoferi* is poorly characterized. In this study, the pathogenicity of a *S. pettenkoferi* clinical isolate from diabetic foot osteomyelitis was compared with a *Staphylococcus aureus* strain in various in vitro and in vivo experiments. Growth kinetics were compared against *S. aureus*, and bacteria survival was assessed in the RAW 264.7 murine macrophage cell line, the THP-1 human leukemia monocytic cell line, and the HaCaT human keratinocyte cell line. Ex vivo analysis was performed in whole blood survival assays and in vivo assays via the infection model of zebrafish embryos. Moreover, whole-genome analysis was performed. Our results show that *S. pettenkoferi* was able to survive in human blood, human keratinocytes, murine macrophages, and human macrophages. *S. pettenkoferi* demonstrated its virulence by causing substantial embryo mortality in the zebrafish model. Genomic analysis revealed virulence factors such as biofilm-encoding genes (e.g., *icaABCD; rsbUVW*) and regulator-encoding genes (e.g., *agr*, *mgrA*, *sarA*, *saeS*) well characterized in *S. aureus*. This study thus advances the knowledge of this under-investigated pathogen and validates the zebrafish infection model for this bacterium.

## 1. Introduction

The genus *Staphylococcus* includes more than 50 species of Gram-positive cocci commonly classified into two categories in human medicine: coagulase-positive staphylococci, mainly represented by *Staphylococcus aureus*, and coagulase-negative staphylococci (CoNS). CoNS are a diverse group of bacteria that range from true non-pathogenic to facultative pathogenic species with variable virulence potential. They share common characteristics involved in the transition to a pathogenic interaction with the host [1]. As CoNS possesses less virulence factors than *S. aureus*, particularly those that cause infection, they are thought to be less harmful. However, CoNS are considered as opportunistic bacteria that colonize healthy people, as well as being the cause of the most common hospital infections, with a growing effect on human health and life [2]. Endogenous infections, such as bacteremia, endocarditis, osteomyelitis, pyoarthritis, peritonitis, mediastinitis, prostatitis, and urinary tract infections, are caused by CoNS present on the host’s skin and mucous membranes [2]. These infections have been linked to over a dozen of the 50 CoNS *Staphylococcus* species that have been identified, including *S. capitis*, *S. chromogenes*, *S. cohnii*, *S. epidermidis*, *S. haemolyticus*, *S. hominis*, *S. lentus*, *S. lugdunensis*, *S. saprophyticus*, *S. sciuri*, *S. simulans*, *S. warneri*, and *S. xylosus* [3]. Nevertheless, other rarer species have been reported, such as *Staphylococcus pettenkoferi* [4]. Moreover, the therapeutic and prophylactic overuse of antibiotics contributes to the increase in infections due to multidrug-resistant CoNS [2].

*S. pettenkoferi* is a comparatively recently identified CoNS, for which its natural habitat, clinical implications, detection, and epidemiology remain to be characterized [5]. Trülzsch et al., who first identified and described this pathogen in Germany in 2002, used 16S ribosomal ribonucleic acid (rRNA) sequencing, biochemical, and physiologic approaches to distinguish it from other CoNS [4]. *S. pettenkoferi* bacteremia was initially described in a 25-year-old tuberculosis patient with extrapulmonary tuberculosis [6]. Since then, around ten case reports of *S. pettenkoferi* bacteremia and one case of osteomyelitis from various countries have been published [6,7,8,9]. Moreover, some *S. pettenkoferi* isolates have been detected as being multidrug resistant to commonly used antibiotics such as beta-lactams, fluoroquinolones, and macrolides, complicating the treatment options [10]. 

Diabetic foot ulcers (DFU) represent one of the most significant complications for patients living with diabetes mellitus. These chronic wounds are responsible for frequent infections spreading to soft tissues and bone structures, constituting diabetic foot osteomyelitis (DFOM), leading to increased amputations, mortality, and morbidity [11]. Whilst the infected DFU and DFOM are mainly polymicrobial [12], Gram-positive cocci, particularly *S. aureus* and CoNS, are the most frequent bacteria isolated [13]. Recently, we have started to observe an emergence of *S. pettenkoferi* isolated from bone biopsies in DFOM [14]. Despite *S. pettenkoferi* being increasingly isolated in human pathology, its pathogenicity remains unexplored. Therefore, in order to evaluate the role of this bacterium as a pathogen, more exploratory investigations are needed. In this study, we demonstrate for the first time that *S. pettenkoferi* can persist in both macrophages and non-professional phagocytes, as well as in human blood, and that its virulence was determined in the zebrafish model of infection. 

## 2. Results

### 2.1. S. pettenkoferi Growth and Biofilm Formation

**Figure 1:**
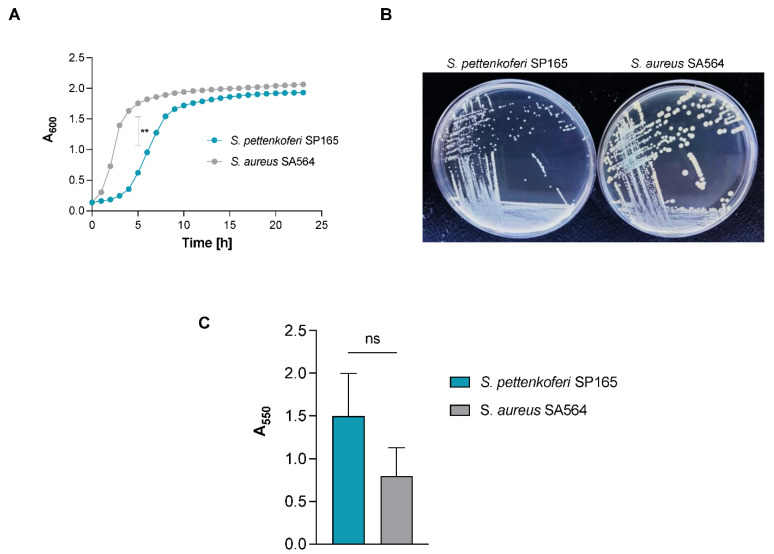
Growth of *S. pettenkoferi* SP165. (**A**) Growth kinetics of *S. pettenkoferi* SP165 (blue circles) and *S. aureus* SA564 (grey circles) strains in TSB. At a culture to flask volume of 1:10, cells were grown at 37 °C and 225 rpm. At each time point (*n* = 3), the data show the mean A_600_ readings SD. Welsh’s *t* test, ** *p* < 0.01. (**B**) Growth of *S. pettenkoferi* SP165 (left panel) compared with *S. aureus* SA564 (right panel) phenotype on TSB agar plate grown at 37 °C for 48 h. (**C**) Kinetics of the complete biofilm formation determined by crystal violet experiment. The optical density (500 nm) is directly linked to the biofilm formation. Means and standard errors for six independent replicates are presented. Statistical differences were obtained by Mann–Whitney; ns, not significant.

*S. pettenkoferi* SP165 and *S. aureus* SA564 grown in vitro followed a typical growth cycle (Figure 1A). A significant difference in exponential growth phase was observable between the two strains (*p* < 0.01) in the first 8 h of growth, with *S. pettenkoferi* SP165 growing slower than *S. aureus*. The corresponding generation time (G = ln2/µmax) was significantly different between *S. pettenkoferi* SP165 and *S. aureus* SA564: 74 min vs. 48 min for *S. aureus* SA564 (*p* < 0.01) (Figure 1A). Furthermore, the differing growth patterns of the two isolates were represented in their colony size at 48 h on TSB agar plates, with *S. pettenkoferi* SP165 having a smaller colony size than *S. aureus* SA564 (Figure 1B). The *S. aureus* SA564 strain produces the carotenoid pigment staphyloxanthin responsible for the characteristic golden color of *S. aureus*, while *S. pettenkoferi* did not present any pigmentation after 72 h of incubation (data not shown). Next, we determined the biofilm formation ability of *S. pettenkoferi* SP165 and *S. aureus* SA564 grown in BHI by the crystal violet microplate-based model. Both strains were able to form biofilms after 48 h of static growth at 37 °C (Figure 1C). Interestingly, *S. pettenkoferi* SP165 produced at least the same amount of biofilm as the *S. aureus* SA564 strain, despite having a slower growth rate.

### 2.2. S. pettenkoferi Persists within Murine and Human Macrophages

**Figure 2:**
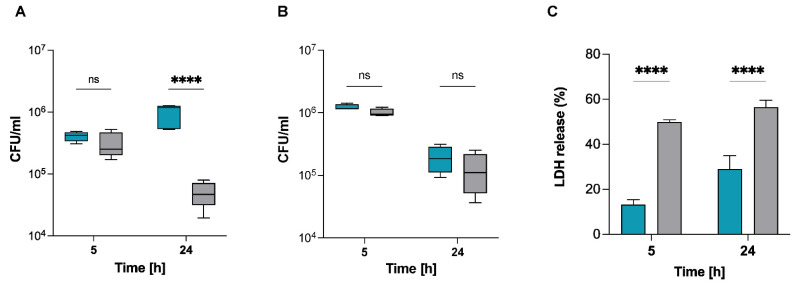
*S. pettenkoferi* SP165 survival in infected macrophages. *S. pettenkoferi* SP165 (blue) and *S. aureus* SA564 (grey) bacteria were used to infect RAW 264.7 (**A**) or THP-1 (**B**) macrophages. The amount of bacteria after 5 and 24 h pGt was evaluated after infection at a multiplicity of infection (MOI) of 20. The average and standard deviation (SD) of five different experiments are represented. (**C**) LDH release was measured using the CyQUANT assay kit after RAW 264.7 macrophage cells were infected at a MOI of 20 for 5 and 24 h, with the exception that the cells were seeded in a 96-well plate and that the extracellular bacteria were not eliminated. Data are expressed relative to the 100% positive control (*n* = 3 biological repeats). A two-way ANOVA test was used to establish statistical significance, with **** *p* < 0.0001; ns, not significant.

Macrophage immune cells have a key role in the eradication of bacteria via phagocytosis [15]. To explore the interaction of *S. pettenkoferi* with macrophages, infection assays were conducted in the murine macrophage cell line RAW 264.7 and primary human THP-1 macrophages. Macrophages were allowed to phagocytose *S. pettenkoferi* SP165 or *S. aureus* SA564, and viable bacteria were recovered at 5 h and 24 h pGt. *S. aureus* SA564 and *S. pettenkoferi* SP165 were able to persist in both macrophage cell lines over time (Figure 2). Interestingly, intracellular *S. aureus* SA564 persisted without replication while *S. pettenkoferi* SP165 was able to replicate within 24 h pGt in RAW 264.7 macrophages (Figure 2A). Our data show that both macrophage cell types failed to kill intracellular *S. pettenkoferi* SP165, but only human THP-1 cells restricted *S. pettenkoferi* SP165 bacterial growth (Figure 2B). In addition, we tested whether the increased survival of *S. pettenkoferi* SP165 could be associated with a decreased cytotoxicity of the infected macrophage cells by using the lactate dehydrogenase (LDH) assay. The LDH assay is a well-established method of detecting necrotic cell death since it measures cellular plasma membrane damage by the release of LDH enzyme into the culture medium. Infection with *S. aureus* SA564 and *S. pettenkoferi* SP165 increased LDH release in culture supernatants over time in infected Raw 264.7 macrophages, but *S. pettenkoferi* SP165 showed significant decreased levels of LDH (Figure 2C). Overall, these findings suggest that, in comparison with *S. aureus*, *S. pettenkoferi* SP165 has a distinct intracellular fate in which the bacteria can survive for long periods of time within macrophages with the induction of lower cytotoxicity. Therefore, the failure of macrophages to eradicate intracellular *S. pettenkoferi* may be a significant deficiency of host innate immunity, allowing for the existence of intracellular reservoirs of viable *S. pettenkoferi*.

### 2.3. S. pettenkoferi Persists in Human Keratinocytes

**Figure 3:**
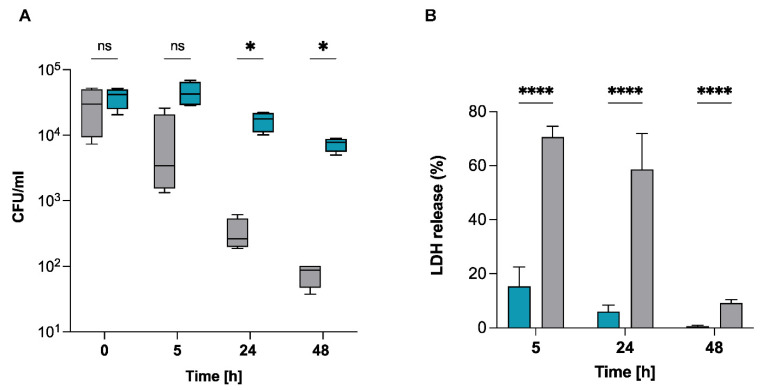
Survival capacity of *S. pettenkoferi* SP165 in non-professional phagocytes. (**A**) At a MOI of 100, bacteria from *S. pettenkoferi* SP165 (blue) and *S. aureus* SA564 (grey) were utilized to infect cells of the keratinocyte cell line HaCaT, and the cells were co-cultured for 90 min. Washing and lysostaphin/gentamicin treatment were used to eliminate extracellular and adhering bacteria, and infected cells were grown for up to 48 h in cell culture media supplemented with lysostaphin. Eukaryotic cells were lysed, and surviving bacteria in lysates were evaluated by counting CFUs at 5, 24, and 48 h after lysostaphin/gentamicin treatment. Percentage survival = (#CFUfinal/#CFUinput) ∗ 100. (**B**) LDH release was measured using the CyQUANT assay kit after HaCaT cells were infected at a MOI of 100 for 5, 24 and 48 h, with the exception that the cells were seeded in a 96-well plate, and the extracellular bacteria were not eliminated. Data are expressed relative to the 100% positive control. Statistical significance was determined by two-way ANOVA test where ns, non-significant; * *p* < 0.05; **** *p* < 0.0001. *n* = 4 biological repeats.

*S. pettenkoferi* was identified in a case of osteomyelitis in a diabetic foot infection, indicating that this strain appears capable of infecting and persisting in non-phagocytic cells such as osteoblasts or epithelial cells [14]. Invasion of non-phagocytic host cells by *S. pettenkoferi* could be an effective mechanism to prevent elimination and maintain infection [2]. To test this hypothesis, human skin keratinocytes were used to evaluate the survival capacity of *S. pettenkoferi* SP165. HaCaT is a non-transformed human keratinocyte line that has been used to investigate epithelial infection [16], cytotoxicity [17], biofilm formation [18], and skin cancer [19]. It can also be employed in models of skin wound infection where the stratum corneum is disrupted and bacteria come into close contact with living keratinocytes [18]. Therefore, we performed invasion experiments to address the persistence of *S. pettenkoferi* SP165 since *S. aureus* has been demonstrated to proliferate and persist intracellularly in HaCaT cells [20,21]. A significant reduction in *S. aureus* SA564 intracellular bacteria compared with *S. pettenkoferi* SP165 was observed over 48 h in HaCaT cells (Figure 3A). Infection with *S. aureus* SA564 increased LDH release in infected keratinocytes over a time-course of 48 h, whereas *S. pettenkoferi* SP165 showed significant decreased levels of LDH in culture supernatants over time (Figure 3B). Taken together, these findings demonstrate that *S. pettenkoferi* SP165 is able to survive intracellularly at late time post-infection in keratinocytes without host cell-induced toxicity.

### 2.4. S. pettenkoferi Avoids Whole Blood Killing but Is Sensitive to Plasma

**Figure 4:**
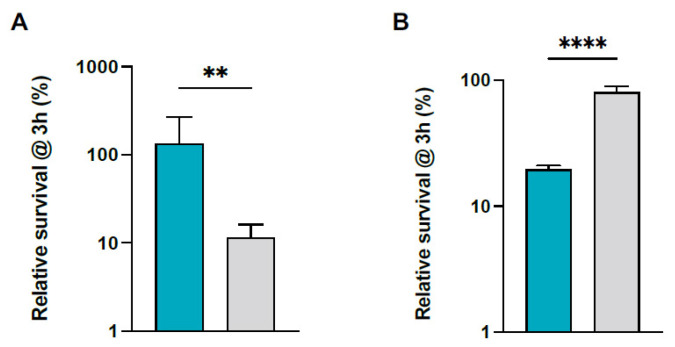
*S. pettenkoferi* SP165 and *S. aureus* SA564 survival in whole blood. Percentage survival of *S. pettenkoferi* SP165 (blue) and *S. aureus* SA564 (grey) in (**A**) freshly collected human whole blood and (**B**) human plasma. Bacteria were cultivated overnight, diluted, inoculated in blood or human plasma, maintained for 3 h at 37 °C, and rotated. Percentage survival = (CFU_timepoint_/CFU_initialinput_) ∗ 100. The data are the average with standard deviation of five separate experiments. ** *p* < 0.01; **** *p* < 0.0001 (Mann–Whitney U test).

Ex vivo survival of *S. pettenkoferi* in human blood was evaluated to define the pathogen’s capacity to induce disseminated infection. After 3 h of incubation in human blood, 100% of the *S. pettenkoferi* SP165 inoculum survived, but only 10% of the *S. aureus* SA564 inoculum survived (Figure 4A). Interestingly, despite a slower growth rate for *S. pettenkoferi* SP165 in early growth (Figure 1), *S. pettenkoferi* SP165 showed around a 10-fold higher rate of survival in blood than *S. aureus* SA564. We next tested the bacteria’s ability to deal with bactericidal effectors found in plasma (Figure 4B). Surprisingly, after 3 h of incubation in plasma, 80% of the *S. aureus* SA564 inoculum survived, but only 20% of the *S. pettenkoferi* SP165 inoculum survived (Figure 4B).

### 2.5. A Bath Infection Model Using Healthy and Wounded Zebrafish Embryos Was Used to Evaluate S. pettenkoferi Pathogenicity

**Figure 5:**
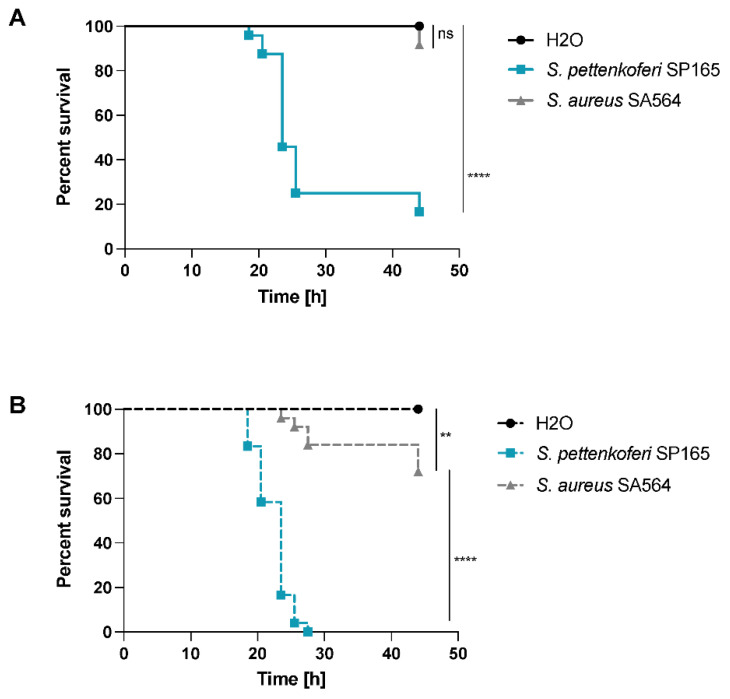
Virulence of *S. pettenkoferi* SP165 and *S. aureus* SA564 strains in zebrafish embryos. Kaplan–Meier representation of the survival of (**A**) healthy zebrafish embryos or (**B**) zebrafish embryos injured in the tail fin at 48 h post-fertilization (hpf) in a bath infected with *S. pettenkoferi* SP165 or *S. aureus* SA564 strains at 2.10^7^ CFU/mL grown in exponential phase or “fish water” (negative control). The proportion of surviving embryos (*n* = 24 for each, indicative of three separate experiments) is used to express the results. Significant difference at ** *p* < 0.01, **** *p* < 0.0001, or no significant difference (ns).

The zebrafish (Danio rerio) is being studied for its ability to mimic human diseases caused by bacterial pathogens [22]. It is a common model for studying host–pathogen interactions [23]. Microinjecting *S. aureus* bacteria into the bloodstream of 1- or 2-day-old post-fertilization (dpf) embryos is a frequent method of generating *S. aureus* infections [24]. Acute infection and death result when the number of bacteria injected exceeds the embryo’s phagocytic capability. To avoid the time-consuming microinjection step, we employed a robust and efficient bath immersion model for *S. pettenkoferi* infection in zebrafish embryos. This innovative approach, which uses wounded embryos to measure bacterial pathogenicity, has already been validated for other pathogens [25,26]. Two separate models were employed for bath immersion infections: immersion alone and immersion following injury, in which the animals’ tail fin tips were wounded prior to infection. The same bacterial solution at the same concentration was used to infect healthy and wounded larvae in a parallel experiments. Bath immersion was first performed on healthy embryos at 2 days post-fertilization, while the mouth was still closed [27]. The viability of zebrafish embryos was monitored for 48 h, and no deaths were recorded with S. aureus SA564, while less than 20% of embryos survived 24 h post infection (hpi) following *S. pettenkoferi* SP1165 infections (Figure 5A). In injured larvae infected with *S. pettenkoferi* SP165, mortalities began at 18 hpi and increased up to 100% by 30 hpi, while embryos infected with *S. aureus* SA564 showed a mortality around 30% by the end of the assay (Figure 5B). There was no mortality among healthy or wounded larvae treated with fish water at the end of the studies (control group). Therefore, our results confirmed the use of this model to study *S. pettenkoferi* virulence.

### 2.6. Whole-Genome analysis of S. pettenkoferi SP165

**Figure 6:**
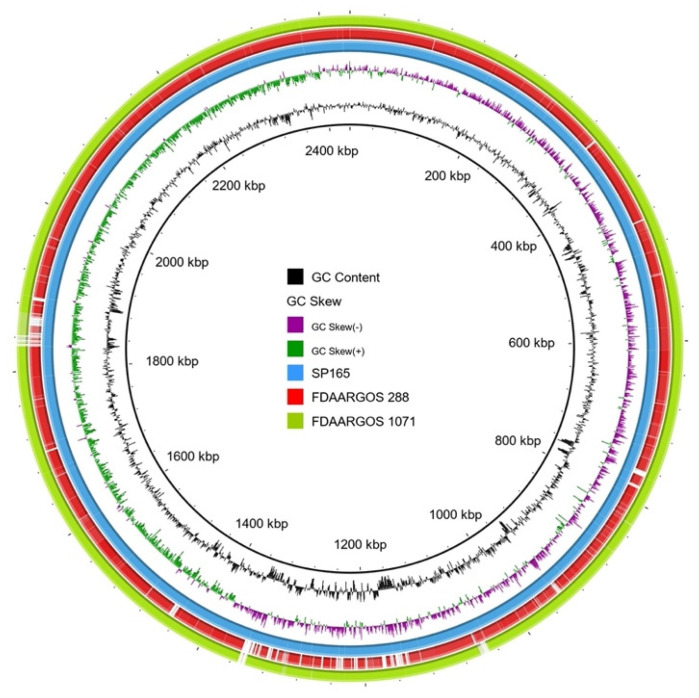
BRIG analysis of *S. pettenkoferi* genomes. The SP165 strain was compared against the two genomes previously described in the literature. The inner blue ring represents the SP165 genome; the middle red one shows the FDAARGOS 288 genome after BLASTn match; while the outer green ring corresponds to FDAARGOS 1071. Only regions with >90% nucleotide identity are colored. Lower identity percentage or no match are represented by blank spaces in each ring. The inner bicolor ring corresponds to the GC skew of *S. pettenkoferi* SP165 isolates. Green profile indicates overabundance of GC nucleotides, whereas purple shows the opposite. The inner black ring represents the GC%.

The virulence potential of SP165 was investigated by whole-genome sequencing. The genome size of *S. pettenkoferi* genomes corresponded to 2,435,720 base pairs (bp), a smaller genome size compared with the two other sequenced strains (Table 1). The *S. pettenkoferi* strains exhibited a similar GC content, with 38.91%, 38.85%, and 39.14% for SP165, FDAARGOS 288, and FDAARGOS 1071, respectively. The numbers of coding DNA sequences (CDS) were 2298 for SP165 compared with 2380 for FDAARGOS 288 and 2318 for FDAARGOS 1071. BRIG-based analyses are shown in Figure 6. The origin of replication was estimated at 1,409,866 bp. The genomic comparison between the *S. pettenkoferi* SP165 and the two strains, FDAARGOS 288 and FDAARGOS 1071, showed a good coverage between each genome, with a high identity (90% coverage and 96.75% and 99.92% identity, respectively). Different virulence factors were identified, detecting some well-known biofilm- encoding genes (e.g., *icaABCD; rsbUVW*) or regulator-encoding genes (e.g., *agr*, *mgrA*, *sarA*, *saeS*) present in *S. aureus* (Table 1). For resistome analysis, the detected mechanisms of resistance corroborated the antibiogram results with methicillinoresistance (*mecA*), fluoroquinolones resistance (mutations in *gyrA* and *gyrB*), macrolides resistance (*msrAB*), rifampicin resistance (*rpoB*, *rpoC*), and fosfomycin resistance (*fosB*) markers.

## 3. Discussion

*S. pettenkoferi* was first identified in 2002 [4] and is part of the normal skin microbiota; however, this bacterium can cause infection, with several cases of severe infection reported [6,7,8,9]. However, to date, *S. pettenkoferi* pathogenic processes and virulence have not been described. Here, we report that *S. pettenkoferi* SP165 isolated from DFOM is a slow-growing species that can either persist in both macrophages and non-professional phagocytes, as well as in human blood. Moreover, its virulence, investigated for the first time in the zebrafish model of infection, was clearly demonstrated. 

In this study, *S. pettenkoferi* SP165 was characterized by a slower exponential growth phase in planktonic growth correlated with a late colony appearance time. Bacterial fitness is generally measured by the rate at which bacterial cells multiply when grown in well-aerated, nutrient-rich medium. Bacteria potential to outcompete their opponents by rapidly expanding must be evaluated against the threat of depleting available nutrients, meaning a prolonged time of starvation. Faster-growing cells, on the other hand, displayed not only a larger number than their rivals, but they might be more resistant against antibiotics according to Gutierrez et al. results [28]. New experimental evolution investigations and a detailed examination of bacterial survival mechanisms have helped researchers to figure out whether pathogen growth is connected to pathogenicity [28]. Numerous mathematical models have been proposed over the last 30 years to characterize the link between rate of growth and virulence heterogeneity [29]. One of the basic ideas in such models is that a link exists between growth rate and pathogenicity, with greater growth rates enhancing dissemination but also promoting host mortality. However, Legget et al. showed that slow-growing pathogens were a lot more virulent than fast-growing pathogens, and that pathogens that breathed or infected via cutaneous lesions were far more virulent than those swallowed [29]. More recently, poor correlations between virulence measures and replication rates suggest that, in addition to growth rate, other characteristics could account for the variations in virulence, such as the potential to control the host immune system [30]. Therefore, the relatively slow growth rate of *S. pettenkoferi* SP165 compared with *S. aureus* SA564 could be an advantage that persists during host infection. In addition, we identified biofilm-encoding genes in the *S. pettenkoferi* SP165 genome and confirmed its ability to form biofilm. As a critical CoNS pathogenicity factor is their ability to form biofilm, these data confirm the clinical relevance of *S. pettenkoferi* SP165 [31].

Furthermore, we established that *S. pettenkoferi* SP165 may survive and proliferate in professional phagocytes as well as in murine macrophages. The death of bacteria upon phagocytosis is due to the collaborative effort of immunological factors, involving NADPH oxidase stimulation and the generation of reactive oxygen radicals [32,33], acidification of phagosomes [34], peptidoglycan hydrolysis mediated by lysozymes, and activation of lysosomal proteases [35]. Despite this, previous in vitro and in vivo studies have demonstrated that *S. aureus* may survive in the phagolysosomes of macrophages, where it begins multiplying. Recently, it was demonstrated that *S. lugdunensis*, a CoNS bacterium that can cause significant infection, has the ability to bypass host immunity. Flannagan et al. showed that ingested *S. lugdunensis* are not killed by macrophages and that the bacteria can survive in mature phagolysosomes for long periods without multiplying [36]. Conversely, *S. pettenkoferi* intracellular persistence might represent a survival strategy for preventing intoxication of the host cell. In the absence of alternative evading processes, this might hypothetically enable internalized *S. pettenkoferi* to avoid extrinsic immunological factors that could result in bacterial killing. The murine and human macrophages were not able to eradicate phagocytosed *S. pettenkoferi*, showing that this pathogen is able to evade the antibacterial properties of phagolysosomes, whereas the mechanisms remain unknown. Furthermore, *S. pettenkoferi* SP165’s failure to grow inside human macrophages varies markedly from that of murine immune cells, highlighting the functional distinctions between RAW 264.7 and primary human macrophages. RAW264.7 macrophages lack a functional caspase-1 inflammasome activity [37,38], potentially contributing to this replication difference, as this pathway is critical for bacterial clearance despite *S. aureus* having developed many strategies for regulating cell death processes such as apoptosis, necroptosis, and pyroptosis in order to generate infection [39]. Furthermore, *S. pettenkoferi*’s ability to survive inside macrophages could be critical in systemic infections in which Kupffer cells remove staphylococci from blood [40,41]. Kupffer cells rapidly remove *S. aureus* from the bloodstream and, due to their inability to eradicate phagocytosed *S. aureus*, provide this pathogen with a secure intracellular habitat in which to grow [40,42]. However, it remains to be determined whether *S. pettenkoferi* can survive in Kupffer cells. Moreover, we demonstrated *S. pettenkoferi* SP165’s ability to persist in non-professional phagocytes such as human keratinocytes without replicating or being outwardly toxic to the host cells. Therefore, our data support the notion that macrophages’ or keratinocytes’ capacity to limit *S. pettenkoferi* intracellular growth is undeniably anti-bacterial; yet, the inability to eliminate ingested cocci is a weakness that might enable *S. pettenkoferi* to remain within the human body, using macrophages as bacterial reservoirs. However, the mechanisms involved in intracellular survival of *S. pettenkoferi* remain to be established.

CoNS including *S. pettenkoferi* are becoming more widely recognized as a primary cause of bacteremia, particularly in patients with medical implants, such as intravenous catheters, artificial heart valves, and joint prosthetics, or those who are immunodeficient [6,43]. In the case of *S. aureus*, its ability to thrive in the human body is dependent on a delicate balance between its various virulence factors and the presence of different host defense mechanisms. *S. aureus* can breach the epithelial and endothelial barriers to reach the bloodstream, regardless of the original location of infection [44,45]. The bacteria come into contact with the innate immune system in the blood, which is made up primarily of neutrophils, monocytes, and the complement system. In this study, the antimicrobial properties of whole human blood were examined towards *S. pettenkoferi* SP165 and *S. aureus* SA564 strains, revealing that *S. pettenkoferi* possesses an approximately 10-fold higher rate of survival than *S. aureus* SA564. However, *S. pettenkoferi* possesses an approximately 4-fold lower rate of survival than *S. aureus* SA564 in human plasma, suggesting that *S. pettenkoferi* is sensitive to bactericidal effectors present in plasma. One hypothesis could be that when *S. pettenkoferi* is not intracellularly protected, as in whole blood cells upon phagocytosis, the human plasma bactericidals are effective. Their role in the pathogenicity of *S. pettenkoferi* remains to be investigated. Moreover, while CoNS do not possess the coagulase that is considered as an important virulence factor for blood survival, they are often associated with bacteremia [2]. However, coagulase binds to host prothrombin and forms staphylothrombin, which activates thrombin protease activity. Regardless of the fact that coagulase is considered to help protect bacteria from phagocytic and immunological responses by causing localized coagulation, its involvement in pathogenicity is unknown [46]. In the case of the *S. epidermidis* CoNS, the induction of coagulase expression dramatically reduced its survival in the blood, suggesting that coagulase synthesis in the blood could be unfavorable to survival [46]. This contradicts the theory that coagulase protects bacteria from immune defenses by producing localized coagulation. It remains to be determined whether the extraordinary capacity of *S. pettenkoferi* to survive in whole human blood is linked to the absence of the coagulase or other mechanisms such as the ability to evade immune clearance, as seen in *S. epidermidis* [47,48]. In addition, the genome of this bacterium revealed high homology with *S. aureus* concerning the presence of virulence factors and the main regulators of this staphylococcal virulence. Their role in the pathogenicity of *S. pettenkoferi* must be elucidated. 

Moreover, to examine *S. pettenkoferi* pathogenicity, we employed a newly established procedure for infection of zebrafish embryos. The zebrafish model offers a number of benefits compared with mammalian infection models related to technical, economic, and ethical issues. Zebrafish are vertebrates, which are genetically and physiologically closer to humans than invertebrate models, and they have a functioning innate immune system in embryos [49]. Because both macrophages and neutrophils play a role in preventing *S. aureus* development, zebrafish have improved our comprehension of *S. aureus* mechanisms developed to avoid the host innate immunity [50], but some can operate as an immune bottleneck, preserving a subpopulation of bacteria from being killed by host cells and causing disseminated infection [51,52]. Interestingly, zebrafish infection studies demonstrated how interactions with commensals on human skin might increase *S. aureus* colonization [53]. In most cases, bacterial infections in zebrafish embryos are performed by microinjection [26]. *S. pettenkoferi* is a bacterium that enters via skin wounds [2]; thus, we applied a bath infection model that mimics real infection. We showed that when healthy or wounded embryos were submerged in *S. pettenkoferi* bacteria at 2 dpf (a developmental time when the mouth is not yet open), significant mortality was reported, demonstrating *S. pettenkoferi* virulence. Surprisingly, even at 2 dpi, *S. pettenkoferi* is virulent, indicating that without mouth ingestion, this pathogen is able to induce mortality in contrario to *S. aureus* SA564. Fin excision has previously been used as a model of “sterile” wounding damage and inflammation [54]. An inflammatory response is triggered by the recruitment of neutrophils and macrophages following an injury, and the embryonic zebrafish fin is also remarkably regenerative [55]. Therefore, *S. pettenkoferi* seems able to manipulate the immune host response to avoid killing in this infection model. Interestingly, while zebrafish cannot replace other vertebrate models such as mice, they can disclose key principles in *S. pettenkoferi* virulence and host defense, and thus support the development of new treatments for staphylococcal diseases. 

## 4. Materials and Methods

### 4.1. Bacterial Strains, Media, and Growth Conditions

The bacterial strains used in this study are listed in Table 2. *S. pettenkoferi* SP165 was isolated from bone biopsies in a DFOM present in a 57-year-old man with type-2 diabetes *mellitus* in the Gard-Occitanie Diabetic Foot Clinic (University hospital of Nîmes, France). *Staphylococcus* strains were plated on Tryptic Soy Agar (TSA) or were grown in Tryptic Soy Broth (TSB) medium at 37 °C and 225 rpm, or were grown in Brain Heart Infusion medium (BHI) for biofilm assays. A microplate reader (Tecan, Lyon, France) was used to monitor bacterial growth in 96-well plates.

### 4.2. Macrophage Culture and Infection

The murine macrophage cell line RAW 264.7 (mouse leukemic monocyte macrophage, ATCC TIB-71) was grown in Dulbecco’s modified Eagle’s medium (DMEM) (Thermo Fisher Scientific, Dreieich, Germany) enriched with 10% fetal calf serum (Thermo Fisher Scientific) at 37 °C in a humidified environment with 5% CO_2_. The human leukemia monocytic cell line THP-1 [57] was cultured in Milieu Roswell Park Memorial Institute (RPMI) medium (Thermo Fisher Scientific) enriched with 10% fetal calf serum (Thermo Fisher Scientific) in a humidified environment at 5% CO_2_ at 37 °C and differentiated into macrophages as previously described [58]. *S. aureus* SA564 and *S. pettenkoferi* SP165 strains were cultured in TSB medium to the mid-exponential growth phase (OD600 = 0.7–0.9) for macrophage infection. The bacteria were collected for 5 min at 4000 rpm, rinsed in sterile PBS, centrifuged at 10,000 rpm for 4 min, and finally resuspended in sterile PBS. THP-1 cells (1 × 10^6^ cells/mL, in 24-well plates) and RAW 264.7 cells (5 × 10^5^ cells/mL, in 24-well plates) were infected with *S. aureus* SA564 or *S. pettenkoferi* SP165 at a MOI of 20:1 (bacteria/cells) and incubated for 1 h at 37 °C and 5% CO_2_. After that, the cells were washed in PBS once more, and the extracellular bacteria were killed by incubation with gentamicin (100 µg/mL) for 30 min. After gentamicin treatment, macrophages were rinsed twice with PBS (T0), then incubated for 5 h and 24 h in fresh medium with 15 µg/mL lysostaphin for *S. pettenkoferi* SP165 and 5 µg/mL lysostaphin for *S. aureus* SA564. By lysing infected macrophages with 0.1% Triton X-100 in PBS, intracellular bacteria were counted. To estimate the number of colony forming units (CFU), macrophage lysates were serially diluted and plated on TSB agar plates and grown at 37 °C.

### 4.3. Keratinocyte Culture and Infection

HaCaT, a well-known human keratinocyte cell line, is a spontaneously transformed human epithelial cell line from adult skin that retains complete epidermal differentiation potential [59]. HaCaT cells were cultured in DMEM (Thermo Fisher Scientific) enriched with 10% fetal calf serum (Thermo Fisher Scientific), 1× Glutamate (Gibco™), and 0.5% penicillin/streptomycin antibiotics (Gibco™) in a humidified environment at 37 °C and 5% CO_2_. *S. aureus* SA564 and *S. pettenkoferi* SP165 were cultured for infection in TSB medium to the mid-exponential growth phase (OD600 = 0.7–0.9). The bacteria were then collected and resuspended in sterile PBS after centrifugation at 10,000 rpm for 5 min. The HaCaT cells (1 × 10^6^ cells/mL, in 24-well plates) were infected with *S. aureus* SA564 or *S. pettenkoferi* SP165 at the MOI of 100:1 (bacteria/cells) and incubated 1 h 30 at 37 °C and 5% CO_2_. After that, the cells were washed in PBS once more, and the extracellular bacteria were killed by incubation with gentamicin (100 µg/mL) for 60 min. Following gentamicin treatment, macrophages were washed twice with PBS (T0) and then incubated for 5, 24, and 48 h in fresh medium with 15 µg/mL lysostaphin. By lysing infected HaCaT cells with 0.1 % Triton X-100 in PBS, intracellular bacteria were counted. Keratinocyte lysates were serially diluted and plated on TSB agar plates and cultured at 37 °C. The number of bacterial colonies at time post-gentamicin treatment (pGt)/number of bacterial colonies at T0 × 100 percent was used to calculate the survival rate of bacteria.

### 4.4. Cell Viability Assay

The release of LDH was quantified using the CyQuant LDH Cytotoxicity Assay Kit (Thermo Fisher Scientific, RockFord, IL, USA) according to the manufacturer’s instructions. Macrophages and keratinocytes cells were infected at a MOI of 20 and 100, respectively, with the exception that the cells were seeded in a 96-well plate and that the extracellular bacteria were not eliminated. Measure of absorbance was performed with the Tecan apparatus (Tecan, Lyon, France). 

### 4.5. Whole Blood and Plasma Killing Assays

*S. pettenkoferi* SP165 or *S. aureus* SA564 were incubated in freshly drawn blood, using EDTA as an anticoagulant. *S. aureus* SA564 and *S. pettenkoferi* SP165 strains were grown to the mid-exponential growth phase (OD_600_ = 0.7–0.9) in TSB. The bacteria were then resuspended in Roswell Park Memorial Institute medium (RPMI, Gibco) after centrifugation at 10,000 rpm for 5 min. Fresh venous human whole blood was obtained from healthy adult volunteers using EDTA-containing Vacutainer tubes (BD) (Etablissement Français du Sang, Montpellier, France) and inoculated with *S. aureus* SA564 or *S. pettenkoferi* SP165 at a concentration of 5 × 10^6^ bacteria/mL and incubated for 3 h at 37 °C on a rotating wheel. The samples were serially diluted and plated on TSB agar plates, followed by incubation at 37 °C. To compute the proportion of bacteria that survived, (CFUtimepoint/CFUinitialinput) × 100 was used.

For plasma killing experiments, freshly drawn blood using EDTA as an anticoagulant was centrifuged for 10 min at 1000× *g*. The supernatant was collected and filtered through a 0.2 µm membrane before being stored at −80 °C until it was required. Plasma was filtered again after thawing with a 0.2 µm membrane. Bacteria were cultured in plasma at the same concentration as in whole blood experiments (5 × 10^6^ bacteria/mL) and incubated for 3 h at 37 °C on a rotating wheel. The samples were serially diluted and plated on TSB agar plates, followed by incubation at 37 °C. To compute the proportion of bacteria that survived, (CFUtimepoint/CFUinitialinput) × 100 was used.

### 4.6. Infection of Danio Rerio Embryos

*S. aureus* SA564 and *S. pettenkoferi* SP165 strains were grown overnight at 37 °C in TSB. Cultures were diluted 1:20 and grown to the mid-exponential growth phase (OD_600_ = 0.7–0.9) in TSB medium. The bacteria were then centrifuged for 10 min at 4000 rpm and reconstituted in fish water with 60 µg/mL sea salt (Instant Ocean) in distilled water with 4.10^−4^ N NaOH at a concentration of 2 × 10^7^ bacteria/mL. The number of bacteria in the inoculum was assessed by plating onto TSB agar plates after dilution in PBS. Experiments were carried out in fish water at 28 °C with the GAB zebrafish line. Bath immersion infections were performed on embryos dechorionated at 48 hpf. Healthy embryos were put in groups of 24 in a Petri dish with the appropriate bacterial suspension (or fish water as a control) and then distributed individually into 96-well (Falcon) plates containing 200 µL of bacterial suspension (or fish water). Embryos to be injured were put on a Petri plate, anesthetized with 0.28 mg/mL tricaine, and a small transection of the tail fin was conducted under a stereomicroscope (Motic) with a 26-gauge needle to injure the tail fin prior to infection. Following tail transection, groups of 24 injured embryos were put in a Petri dish with the appropriate bacterial suspension (or fish water as a control) and then dispersed individually into 96-well plates (Falcon) containing 200 µL of bacterial suspension (or fish water). The plates were held at a constant temperature of 28 °C during the incubation process (bacteria are kept throughout the experiment in fish water, which does not support *S. aureus* SA564 or *S. pettenkoferi* SP165 growth). The absence of a heartbeat allowed the number of dead embryos to be counted visually.

### 4.7. Crystal Violet Biofilm Assay

Biofilm formation was measured by incubating 200 µL of bacterial cultures in 96-well microtiter plates in BHI after an overnight incubation at 37 °C and a dilution to produce a final optical density of 0.1 at OD600 nm. The plates were incubated for 48 h at 37 °C in humid chamber. After incubation, adherent cells were washed with PBS and air dried, and crystal violet (0.1%) was added for 20 min at room temperature. An amount of 100 µL of acetic acid (33%) per well was added for 15 min to dissolve the biofilm, and the measure of absorbance was performed with a Tecan apparatus (Tecan, Model Spark, Grödig, Austria GmbH) at OD550 nm.

### 4.8. Ethical Statement

All animal experiments were carried out at the University of Montpellier in accordance with European Union recommendations for the care and use of laboratory animals (http://ec.europa.eu/environment/chemicals/labanimals/home en.htm (accessed on 15 March 2021)) and were authorized by the Direction Sanitaire et Vétérinaire de l’Hérault and Comité d’Ethique pour l’Expérimentation Animale under reference CEEALR- B4-172-37 and APAFIS#5737-2016061511212601 v3. Adult zebrafish were not killed for this study, and breeding of adult fish followed the international norms set out by the EU Animal Protection Directive 2010/63/EU. According to the EU Animal Protection Directive 2010/63/EU, all studies were conducted prior to the embryos’ free feeding stage and did not constitute animal experimentation. The cardiac rhythm was used as a clinical criterion for survival graphs. At the end of the survival monitoring, the plates were parafilmed and quickly frozen, stored at −20 °C for 48 h to ensure embryo’s death, and then autoclaved with the bacterial contaminated waste.

### 4.9. Whole-Genome Analysis

*S. pettenkoferi* strain SP165 was cultivated aerobically at 37 °C for 24 h on Columbia sheep blood agar plates (5%) (Becton Dickinson, Le Pont-de-Claix, France). Following the manufacturer’s instructions, genomic DNA was extracted using the DNeasy UltraClean Microbial Kit (QIAGEN). Whole Genome Sequencing (WGS) was carried out using Illumina MiSeq sequencing equipment (Illumina) using paired-end (PE) read libraries (PE250) made with an Illumina Nextera XT DNA Library Prep Kit (Illumina) according to the manufacturer’s instructions. To examine data quality, raw readings were processed using FastQC (v.0.11.9). To eliminate leftover PCR primers and filter low quality bases (Q_score < 30) and short reads (<150 bp), the Cutadapter tool (v.1.16) was used, which was implemented in Python (v.3.5.2). The downstream analysis included the filtered trimmed reads. Using the CLC genomics workbench 7 (Qiagen), reads were mapped to the *S. pettenkoferi* FDAAGOS_288 genome (GenBank accession number: GCA_002208805.2) using default settings; length fraction: 0.5, similarity fraction: 0.8. Shovill v1.1.0 software on the Galaxy platform was used to process the assembled contigs for microbial genome annotation. The CONTIGuator website was used to produce consensus sequences, utilizing *S. pettenkoferi* FDAARGOS_288 as the reference. The virulence factor database (VFDB) was utilized to identify virulence factor-encoding genes from genome sequences (https://www.mgc.ac.cn/VFs/ (accessed on 22 October 2021)) [60]. On assembled genomes, antimicrobial resistance genes were acquired from ABRIcate using the ResFinder database [61,62]. The genome was annotated using PROKKA v1.14.5. The origin of replication was determined using GenSkew software (http://genskew.csb.univie.ac.at/ (accessed on 22 October 2021)), and plasmids were screened using the PlasmidFinder 2.0 webtool (https://cge.cbs.dtu.dk/services/PlasmidFinder/ (accessed on 22 October 2021)). WGS was subjected to other investigations, such as circular genome representation (using the BLAST Ring Image Generator program) (BRIG) [63]). The newly sequenced strain can be found under the BioProject: PRJNA768314 for the raw reads and assembled genomes: SP165 SAMN22027592. 

### 4.10. Statistical Analyses

GraphPad software package Prism 6.01 was used to estimate the statistical significance of differences across groups, which is indicated in the relevant figure legends.

## Figures and Tables

**Table 1:** General characteristics of *S. pettenkoferi* SP165 strain.

Features	SP165	FDAARGOS 288	FDAARGOS 1071
Biosample	SAMN22027592	SAMN06173301	SAMN16357240
Assembly	SHOVILL 1.1.0	CA v. 8.2	SMRT v. 7.1.0, HGAP v. 4
Genome size (bp)	2,435,720	2,502,360	2,449,395
Number contigs	54	ND	ND
GC Content (%)	38.91	38.85	39.14
Number CDS	2298	2380	2318
Year of collection	2021	2014	ND
Year of sequencing	2021	2021	2020
Locality	Nîmes (France)	Washington (USA)	Braunschweig (Germany)

ND, not determined; CDS, coding DNA sequences.

**Table 2:** Strains used in this study.

Strain	Description	Resistance Profile	Reference
S. aureus SA564	S. aureus clinical isolate, wild type	PEN	[56]
S. pettenkoferi SP165	S. pettenkoferi clinical isolate from diabetic foot osteomyelitis, wild type	PEN, OXA, ERY, LIN, OFX, RIF, FOS	This study

PEN, penicillin G; OXA, oxacillin; ERY, erythromycin; LIN, lincomycin; OFX, ofloxacin; RIF, rifampicin; FOS, fosfomycin.

## Data Availability

On reasonable request, the corresponding author will provide the datasets produced and analyzed in the present work.
